# The impact of comorbidity on the quality of life of people who recovered from COVID-19 in Bangladesh

**DOI:** 10.1016/j.ijregi.2024.03.006

**Published:** 2024-03-16

**Authors:** Md. Abdullah Saeed Khan, Koustuv Dalal, Mehedi Hasan, Miah Md. Akiful Haque, Nusrat-E-Mozid, Mosharop Hossian, Tajrin Rahman, Ramisha Maliha, Archi Mutsuddi, Md. Utba Rashid, Mohammad Ali Hossain, Mohammad Hayatun Nabi, Mohammad Delwer Hossain Hawlader

**Affiliations:** 1 Infectious Disease Hospital, Dhaka, Bangladesh; 2 School of Health Sciences, Division of Public Health Science, Mid Sweden University, Östersund, Sweden; 3 Department of Public Health, North South University, Dhaka, Bangladesh; 4 Public Health Promotion and Development Society (PPDS), Dhaka, Bangladesh; 5 Department of Epidemiology and Biostatistics, Arnold School of Public Health, University of South Carolina, Columbia, SC 29208, USA; 6 Ibn Sina Medical College Hospital, Dhaka, Bangladesh

**Keywords:** COVID-19, Comorbidity, Quality of life, Bangladesh

## Abstract

**Objectives:**

COVID-19 affects the quality of life (QoL) in a reverse way after recovery, which might be multiplied by the comorbid non-communicable diseases. This study explored the relationship between comorbidities and the QoL of people who recovered from COVID-19 in Bangladesh.

**Methods:**

The cross-sectional study was conducted among 3244 participants between June 2020 and November 2020 using a pre-tested questionnaire through over-the-phone interviews. The WHOQOL-BREF was used to explore the QoL among the study participants. A multivariable linear regression model was conducted to identify the effects of the number of comorbidities on QoL scores of all four domains.

**Results:**

Of 3244 patients who recovered from COVID-19, over one-third (39.4%) had one or more chronic diseases, such as hypertension, diabetes mellitus, ischemic heart disease, bronchial asthma/chronic obstructive pulmonary disease, chronic kidney disease, and cancer. Around 46.85% of the participants aged above 40 years presented with one to two chronic diseases, and 16.33% had three or more chronic diseases. Among all comorbidities, the participants with cancer and chronic kidney disease were found to have relatively lower scores in all four domains than other comorbidities. The lowest QoL scores were observed in the psychological domain. Those with three or more simultaneous chronic comorbidities had the lowest QoL score in all four domains: physical, psychological, social relationship, and environmental.

**Conclusions:**

Persons who recovered from COVID-19 with comorbidities undergo a lower QoL. Therefore, special attention is required to these vulnerable groups to ensure their smooth recuperation.

## Highlights

• The quality of life (QoL) of people who recovered from COVID-19 is important.
• The effect and interaction of chronic comorbidities on QoL were not fully explored.
• Individuals with chronic comorbidities need more attention to restore QoL.
• Non-communicable comorbidity of patients with COVID-19 lower QoL scores.
• Health services for chronic non-communicable diseases should be strengthened.
• Emphasis on mental health services is an urgent task.

## Introduction

COVID-19 has impacted millions worldwide since its beginning and affected people from all walks of life. People who recover from the disease continue to have its effects on their lives [1]. Many people experience lingering health problems and decreased health-related quality of life (HRQoL) after an acute illness of COVID-19 [1,2]. Studies found that numerous individuals who recovered from the disease reported a worsened HRQoL after 9 months of recovery [3]. Moreover, coexisting diseases may have an additive effect on the recovered patients’ health outcomes [4].

The resurgence and exacerbation of non-communicable diseases (NCDs) present a significant and underexplored aspect of COVID-19 recovery. Owing to the direct and indirect effects of the virus and treatment, persistent cardiac involvement, including hypertension, diabetes, obesity, sleep apnea, and hyperlipidemia, was observed in a cohort of patients with COVID-19 71 days after diagnosis, according to a study [5]. In addition, a variety of symptoms, such as fatigue, shortness of breath, mental fog, chest pain, and headache, can develop with prolonged COVID-19 infections, interfering with daily life [6].

Multimorbidity, in conjunction with social and environmental factors, increases the complexity of care and resource consumption [7]. Studies have shown that chronic diseases frequently impair people's HRQoL to a great extent [8,9]. On the other hand, COVID-19 tends to cause severe critical illness in patients with pre-existing chronic diseases [10]. In addition, older age and the presence of chronic disease are associated with long-lasting COVID-19, the so-called long COVID, in patients [11]. Consequently, it is imperative to investigate the nuanced impact of comorbidities on the various dimensions of quality of life (QoL) among individuals who have recovered from COVID-19.

In the context of Bangladesh, where the prevalence of NCDs is noteworthy, understanding the association between comorbid medical illnesses and post-COVID-19 QoL becomes crucial. Our recent nationwide QoL study conducted in patients who recovered from COVID-19 in Bangladesh found lower QoL in the presence of NCDs [12]. However, there exists a gap in the comprehensive exploration of the effects of chronic comorbidities on the physical, psychological, social, and environmental domains of QoL in this population. Given the escalating importance of prioritizing individuals with multiple coexisting diseases for comprehensive care and rehabilitation, the present study aimed to assess how comorbidities affected the QoL of people who recovered from COVID-19 in Bangladesh.

## Methods

### Study design and participants

This cross-sectional study was conducted among 3244 patients diagnosed with COVID-19, confirmed by reverse transcription-polymerase chain reaction, from June 2020 to November 2020 and then recovered either clinically or by a negative reverse transcription-polymerase chain. The patients receiving treatment for COVID-19 and those who were critically ill, pregnant, or mentally unstable were excluded from the study. We collected a list of patients who tested positive for COVID-19 from the whole country for the purpose of our study. Then, we randomly chose 3244 people from the list (1% of the total infected population at that time point from all over the country). We used the World Health organization Quality of Life Brief Version (WHOQOL-BREF) to assess the QoL among the study participants [13]. The details of the methodology were reported elsewhere [12].

### Study instrument

The pre-formed and pre-tested questionnaire used in our study consisted of three sections. In the socio-demographic section of the questionnaire, we asked about the participant's age, gender, residence, religion, the highest level of education, marital status, and monthly income. In the second section, participants’ hospitalization history due to COVID-19, personal history, and comorbidity profile, including hypertension (HTN), diabetes mellitus (DM), ischemic heart disease (IHD), bronchial asthma (BA)/chronic obstructive pulmonary disease (COPD), chronic kidney disease (CKD), and cancer, were queried. To assess the QoL of patients who recovered from COVID-19, we used a validated Bangla version of the WHOQOL-BREF [13]. The scale explores QoL in four domains: physical, psychological, social relationships, and environment, with each item scored on a 5-point Likert scale.

### Study procedure

We conducted an over-the-phone interview, considering the COVID-19 pandemic situation. Although our study participants came from all over the country, interviewers were assigned based on their location to avoid linguistic obstacles. We took down the responses of individuals who felt at ease taking part in the survey. Furthermore, before the interview, we notified the respondents that there was no right or incorrect answer. Misunderstood items were repeated, and responders were encouraged to interpret the questions in their own way. The scoring of the WHOQOL-BREF questionnaire was graded using the manual's guidelines. [13] The WHOQOL-BREF has solid internal consistency among our respondents (Cronbach alpha = 0.89).

### Statistical analysis

We presented descriptive statistics of the categorical (frequencies and percentages) and continuous variables (mean ± SD) according to the number of chronic diseases. After checking the normality assumption, the chi-square test, independent samples *t*-test, and analysis of variance were used to compare categorical and continuous variables for different groups. The influence of different comorbidities on four QoL dimensions, namely, physical, psychological, social, and environmental, was studied using descriptive and inferential approaches. A multivariable linear regression model was conducted to identify the effects of the number of comorbidities on QoL scores of all four domains of the WHOQOL-BREF scale. The results were reported by beta coefficients and corresponding 95% confidence intervals (CIs). For statistical analysis and graphs, we used STATA (Version 16.1) and RStudio (Version 1.4.1106).

## Results

**Figure 1:**
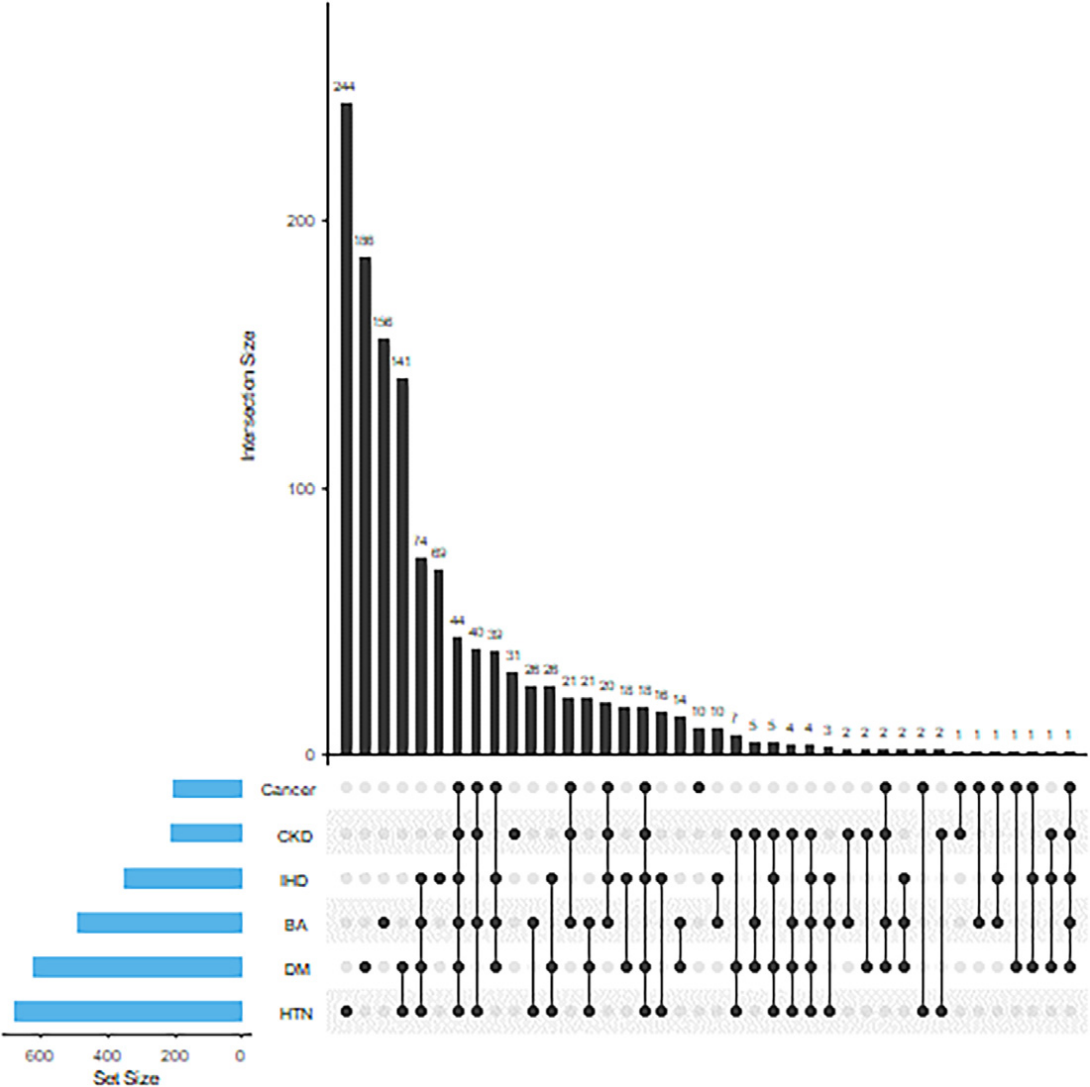
Distribution of chronic disease among participants (isolated and combined).

BA, bronchial asthma; CKD, chronic kidney disease; DM, diabetes mellitus; HTN, hypertension; IHD, ischemic heart disease.

Of the 3244 participants who recovered from COVID-19, 1277 (39.4%) were experiencing one or more chronic diseases, including HTN, DM, IHD, BA/COPD, CKD, and/or cancer. More than one-fourth of the participants had one to two concomitant chronic diseases, and one-tenth of the participants had three or more chronic diseases. HTN, DM, and BA were the three most frequent chronic diseases found among the participants. Concerning the simultaneous presence of multiple chronic diseases, the most common combinations were HTN and DM, followed by a combination of HTN, DM, BA, and IHD (Figure 1).

Table 1 describes the socio-demographic characteristics and their association with chronic diseases among 3244 participants who recovered from COVID-19. Of all participants aged >40 years, nearly one-third had more than one chronic disease. However, the majority of those under the age of 40 years did not have any chronic ailments, and this difference was statistically significant (*P* <0.001). The distribution of chronic diseases was statistically similar across the sexes (*P* = 0.546). Individuals living in the urban areas had more chronic diseases than the other two areas (*P* <0.001). Compared with those who completed secondary school certificate (SSC) / higher secondary certificate (HSC) or upper levels of education, participants with primary or no education had a significantly higher proportion of chronic diseases (*P* = 0.043). Unmarried participants had a higher proportion of chronic diseases than married and other participants (*P* <0.001). In terms of occupation, currently unemployed (including retired) participants had a significantly higher proportion of chronic disease than those who are employed (*P* = 0.001). Participants from the higher-income category had at least one chronic disease than those of lower-income categories (*P* <0.001). Current smokers had significantly more chronic diseases than past and never smokers (*P* <0.001). Participants with a history of admission during COVID-19 illness had a higher proportion of chronic diseases than their counterparts. (*P* <0.001).

**Table 1:** Participant characteristics by number of chronic diseases (n = 3244).

Characteristics	Total	Chronic diseases			*P*-value
		None	1-2	≥ 3	
		(n = 1967)	(n = 932)	(n = 345)	
**Age (years)**	3244	34.39 ± 9.91	44.39 ± 13.19^a^	43.78 ± 14.53^a^	<0.001^b^
**Age groups**					
≤ 40	2117	1552 (73.31)	404 (19.08)	161 (7.61)	<0.001^b^
>40	1127	415 (36.82)	528 (46.85)	184 (16.33)	
**Gender**					
Male	2300	1398 (60.78)	666 (28.96)	236 (10.26)	0.546
Female	944	569 (60.28)	266 (28.18)	109 (11.55)	
**Residence**					
Rural	404	279 (69.06)	107 (26.49)	18 (4.46)	<0.001^b^
Urban	2382	1379 (57.89)	688 (28.88)	315 (13.22)	
Semi-urban	458	309 (67.47)	137 (29.91)	12 (2.62)	
**Education**					
Primary or none	381	211 (55.38)	123 (32.28)	47 (12.34)	0.043^c^
SSC/HSC	1107	673 (60.79)	327 (29.54)	107 (9.67)	
Graduate	1228	776 (63.19)	316 (25.73)	136 (11.07)	
Post-graduate	528	307 (58.14)	166 (31.44)	55 (10.42)	
**Marital status**					
Single	531	428 (80.60)	79 (14.88)	24 (4.52)	<0.001^b^
Married	2642	1523 (57.65)	821 (31.07)	298 (11.28)	
Others	71	16 (22.54)	32 (45.07)	23 (32.39)	
**Occupation**					
Unemployed	122	49 (40.16)	53 (43.44)	20 (16.39)	<0.001^b^
Service holder	1714	1112 (64.88)	438 (25.55)	164 (9.57)	
Other	1408	806 (57.24)	441 (31.32)	161 (11.43)	
**Monthly income (BDT)**					
<20000	746	475 (63.67)	186 (24.93)	85 (11.39)	0.001^b^
20000-40000	1260	780 (61.90)	337 (26.75)	143 (11.35)	
40000-60000	519	308 (59.34)	154 (29.67)	57 (10.98)	
60000+	476	251 (52.73)	173 (36.34)	52 (10.92)	
**History of hospital admission**					
Admitted	838	339 (40.45)	283 (33.77)	216 (25.78)	<0.001^b^
Not admitted	2402	1626 (67.69)	647 (26.94)	129 (5.37)	
**Smoking history**					
Never smoker	1877	1213 (64.62)	537 (28.61)	127 (6.77)	<0.001^b^
Present smoker	809	384 (47.47)	228 (28.18)	197 (24.35)	
Past smoker	558	370 (66.31)	167 (29.93)	21 (3.76)	

a Data were expressed as n (%) or mean (SD), where appropriate; *P*-value was determined by chi-square test and analysis of variance, where appropriate. Pairwise comparisons were done using Tukey (*P* <0.05 compared with none);

b *P* <0.001;

c *P* <0.05.

**Figure 2:**
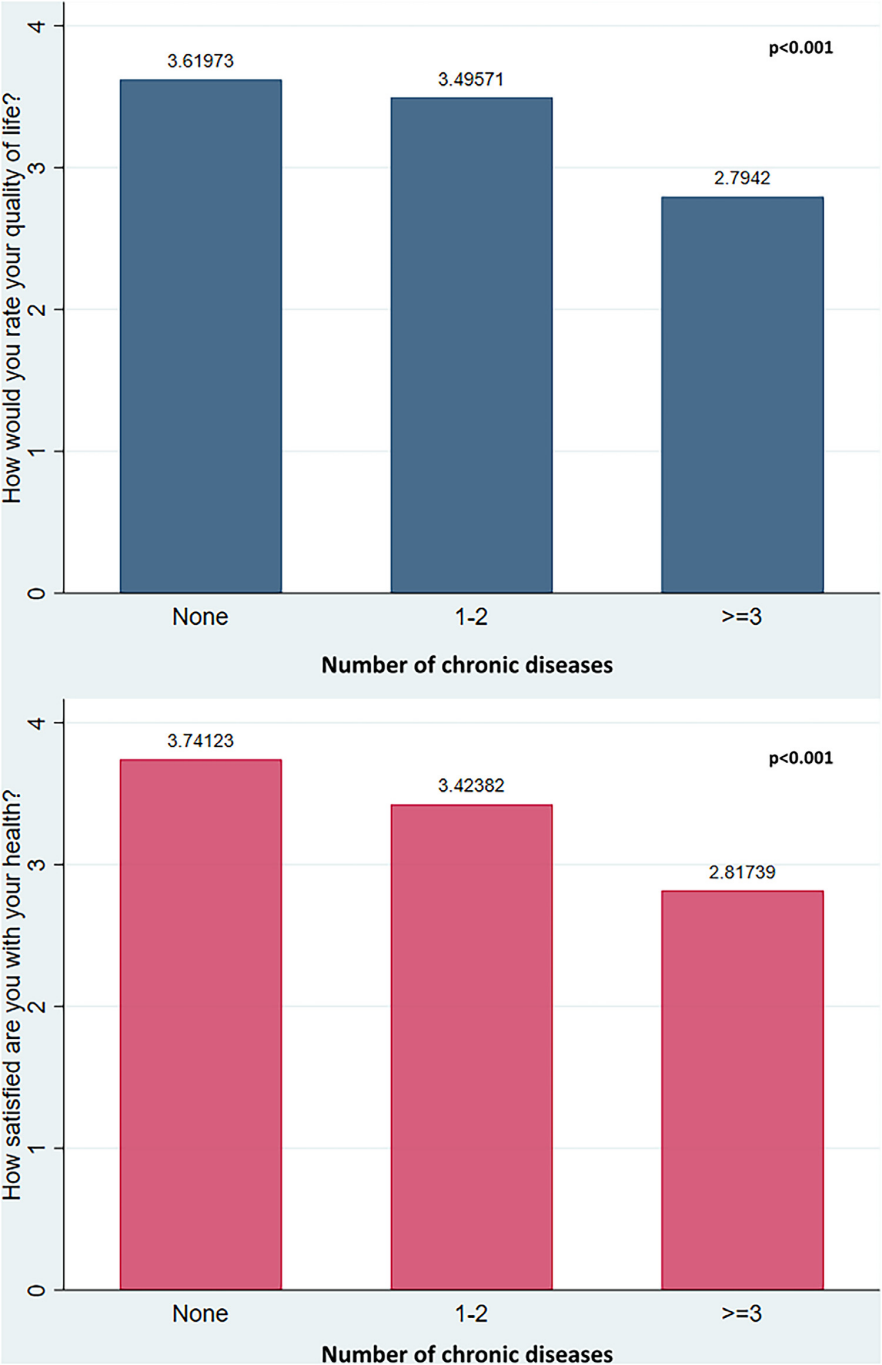
Participants’ perceptions about their quality of life and health categorized by the number of chronic diseases (*P*-values were determined by one-way analysis of variance. Post hoc analysis using Tukey reveals that for all pair-wise comparisons of chronic disease category *P*-value was significant at the <0.001 level).

The WHOQOL-BREF questions 1 and 2 asked about the participants’ overall QoL and physical health assessment. The average QoL rating (q1) was significantly lower among participants with chronic diseases (*P* <0.001). The rating was even lower in participants with three or more chronic diseases than those with one to two chronic diseases. A similar decreasing health satisfaction score (q2) was noted in participants with a higher number of chronic diseases (*P* <0.001) (Figure 2).

**Figure 3:**
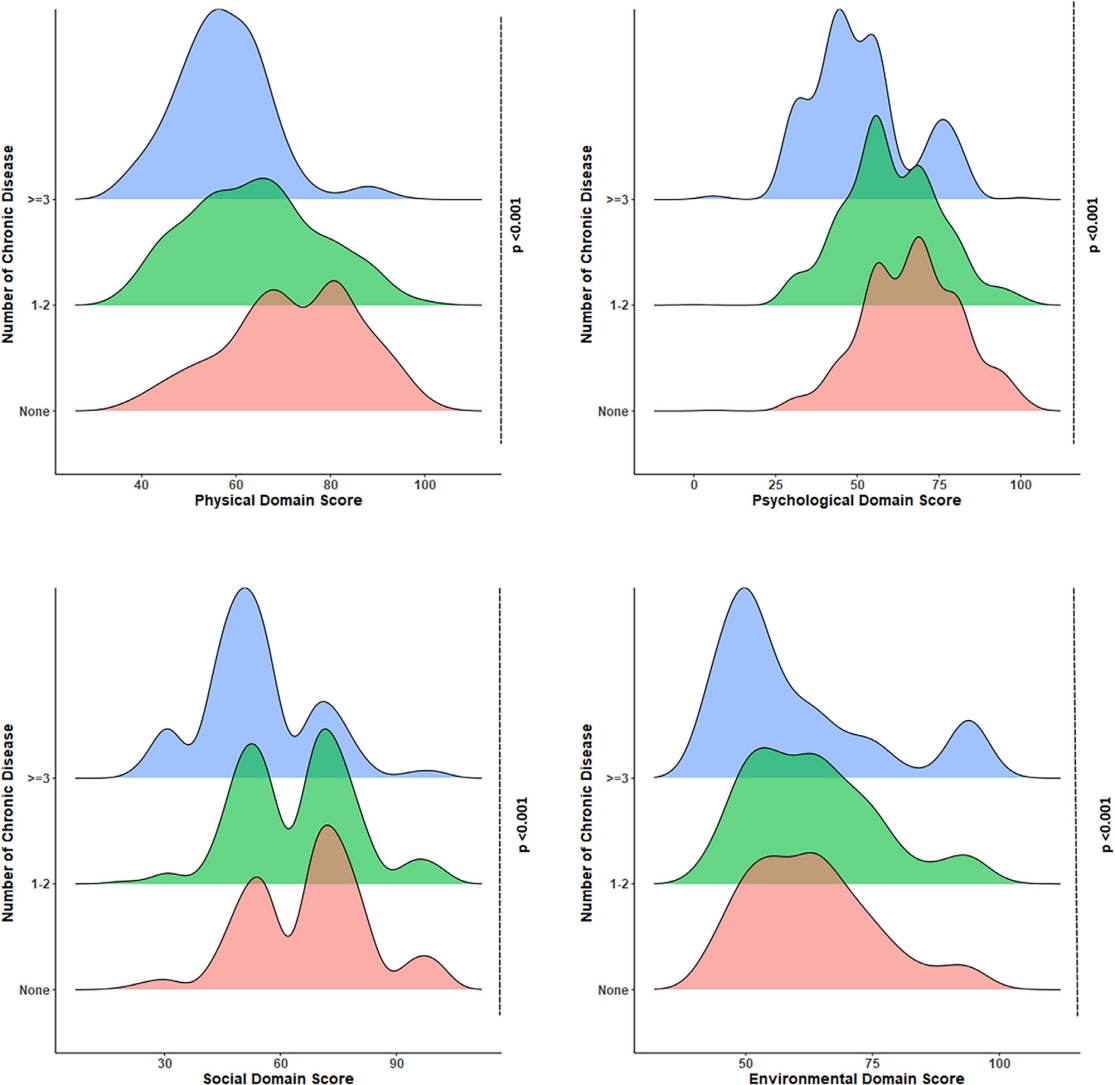
Quality of life scores in physical, psychological, social, and environmental domains by the number of chronic diseases (*P*-values were determined by one-way analysis of variance. Post hoc analysis using Tukey reveals that for all pair-wise comparisons of chronic disease category, the *P*-value was significant at the <0·001 level in all domains, except environmental domain, where the score was statistically similar (*P* >0.05) between participants without chronic disease and participants with one to two chronic diseases).

A ridgeline density plot of overall scores for all disease categories across the four domains of QoL is portrayed in Figure 3. The domain scores showed a uniform distribution in physical health and environmental domains, whereas a relatively broad distribution was noted in the psychological and social relationship domains. Overall, lower scores can be observed in the psychological domain than in the three other domains, emphasizing the higher psychological burden of the participants. Moreover, scores in all four domains were significantly higher in participants with no chronic disease than those with chronic diseases (*P* <0.001 for all domains).

A comparison of QoL scores in the physical, psychological, social, and environmental domains regarding the presence and absence of individual chronic disease showed that the presence of any disease was associated with a significantly lower score in all domains (*P* <0.001), except for IHD and cancer. The latter two were found to be comparable within their groups in the environmental domain (Table 2). However, cancer and CKD were associated with relatively lower scores in all domains than other NCDs.

**Table 2:** Quality of life score of participants at four domains across individual chronic disease.

Chronic diseases	Physical health domain	Psychological domain	Social relationship domain	Environmental domain
**Hypertension**				
Present (n = 683)	61.66 ± 0.51	56.78 ± 0.60	61.03 ± 0.59	61.63 ± 0.53
Absent (n = 2561)	70.00 ± 0.28	65.00 ± 0.29	66.19 ± 0.31	63.07 ± 0.25
*P*-value	<0.001^a^	<0.001^a^	<0.001^a^	0.011^a^
**Diabetes**				
Present (n = 621)	61.13 ± 0.53	56.46 ± 0.61	60.98 ± 0.63	63.77 ± 0.59
Absent (n = 2623)	69.93 ± 0.28	64.89 ± 0.29	66.07 ± 0.31	62.52 ± 0.24
*P*-value	<0.001^a^	<0.001^a^	<0.001^a^	0.034^b^
**Ischemic heart disease**				
Present (n = 354)	58.43 ± 0.59	54.94 ± 0.79	56.97 ± 0.76	62.29 ± 0.82
Absent (n = 2890)	69.44 ± 0.27	64.29 ± 0.28	66.09 ± 0.29	62.82 ± 0.24
*P*-value	<0.001^a^	<0.001^a^	<0.001^a^	0.464
**Bronchial asthma/chronic obstructive pulmonary disease**				
Present (n = 492)	58.73 ± 0.49	53.67 ± 0.68	56.43 ± 0.70	60.68 ± 0.69
Absent (n = 2752)	69.94 ± 0.27	64.99 ± 0.28	66.65 ± 0.29	63.14 ± 0.24
*P*-value	<0.001^a^	<0.001^a^	<0.001^a^	<0.001^a^
**Chronic kidney disease**				
Present (n = 212)	59.72 ± 0.60	53.61 ± 1.12	53.85 ± 0.89	57.46 ± 0.85
Absent (n = 3032)	68.84 ± 0.26	63.95 ± 0.27	65.89 ± 0.28	63.14 ± 0.24
*P*-value	<0.001^a^	<0.001^a^	<0.001^a^	<0.001^a^
**Cancer**				
Present (n = 208)	58.63 ± 0.49	51.30 ± 1.03	51.49 ± 0.89	61.57 ± 1.24
Absent (n = 3036)	68.90 ± 0.26	64.09 ± 0.28	66.03 ± 0.28	62.85 ± 0.23
*P*-value	<0.001^a^	<0.001^a^	<0.001^a^	0.173

*P*-value was determined by independent samples *t*-test;

a *P* <0.001;

b *P* <0.05.

The multiple linear regression analyses demonstrated that after adjusting for socio-demographic characteristics, smoking habits, and history of hospital admission during COVID-19 illness, the presence of chronic diseases in individuals who recovered from COVID-19 were independently associated with a significantly lower QoL score in nearly all four domains (Table 3). Compared with those with no chronic disease, participants with one to two diseases were found to have 6.79 fewer points (95% CI −7.96 to −5.61) in the physical health domain, with 4.37 fewer points (95% CI −5.65 to −3.09) in the psychological score, and 3.03 fewer points (95% CI −4.33 to −1.73) in social relationship domain. Although persons with three or more concomitant chronic diseases had even lower scores in the physical domain (*β* = −11.64, 95% CI −13.38 to −9.89), psychological domain (*β* = −11.08, 95% CI −12.98 to −9.19), social relationship domain (*β* = −11.58, 95% CI −13.51 to −9.66), and environmental domain (*β* = −2.7, 95% CI −4.48 to −1.07).

**Table 3:** Linear regression analysis examining effect of chronic disease on different domains of quality of life.

Domains and chronic disease category	Unadjusted β-coefficient (95% CI)	*P*-value	Adjusted β-coefficient (95% CI)^a^	*P*-value
	**Physical health**			
None	Reference		Reference	
1-2	−8.22 (−9.27 to −7.17)	<0.001	−6.79 (−7.96 to −5.61)	<0.001
≥ 3	−14.88 (−16.42 to −13.33)	<0.001	−11.64 (−13.38 to −9.89)	<0.001
	**Psychological**			
None	Reference		Reference	
1-2	−6.25 (−7.40 to −17.16)	<0.001	−4.37 (−5.65 to −3.09)	<0.001
≥ 3	−15.48 (−17.16 to −13.80)	<0.001	−11.08 (−12.98 to −9.19)	<0.001
	**Social relationship**			
None	Reference		Reference	
1-2	−2.83 (−4.02 to −1.64)	<0.001	−3.03 (−4.33 to −1.73)	<0.001
≥ 3	−13.32 (−15.06 to −11.57)	<0.001	−11.58 (−13.51 to −9.66)	<0.001
	**Environmental**			
None	Reference		Reference	
1-2	0.45 (−0.56 to 1.47)	0.378	0.64 (−0.50 to 1.79)	0.272
≥3	−2.93 (−4.42 to −1.43)	<0.001	−2.7 (−4.48 to −1.07)	0.001

CI, confidence interval.

a Adjusted for age, sex, residence, education, marital status, income, history of admission in hospital, and smoking habit.

## Discussion

Bangladesh is amid an epidemiological shift, with an aging population whose societal, health, and financial concerns must be emphasized. Given the high prevalence of non-communicable chronic diseases in the adult population of Bangladesh [14], this study sought to determine the impact of comorbidities on the different aspects of the QoL of Bangladeshi patients who recovered from COVID-19.

Almost all the six non-communicable comorbid diseases, namely, HTN, DM, IHD, BA/COPD, CKD, and cancer, were responsible for significantly lower QoL scores in the physical health, psychological, social relationship, and environmental domains of life. IHD was associated with the lowest score in the physical health domain, cancer in the psychological and social relationship domain, and CKD in the environmental domain. Overall, cancer was associated with a lower score practically in all four domains, with CKD being the second in terms of impact. The association of IHD with lowered scores in the physical health domain can be attributed to the decline in physical functionality that occurs because of COVID-19’s influence on individuals with pre-existing heart problems. Studies have reported that patients with IHD affected by COVID-19 are more susceptible to experiencing exacerbated heart conditions and myocardial injury and requiring mechanical ventilation [15,16]. Patients with cancer continuously live under the pressure of physical, psychological, and emotional distress [17] and lead a life with a significant reduction in quality [18]. Therefore, concomitant COVID-19 could have adversely affected our participant's suppressed physical and mental well-being. On a similar note, patients with CKD, particularly, those undergoing repeated hemodialysis, often have depression, anxiety, cognitive decline, and a low QoL [19]. A recent study in patients with CKD in Bangladesh found a decreased QoL in 96.7% of the patients [20]. Therefore, a further reduction in the QoL score in a patient with CKD who recovered from COVID-19 is conceivable because of the disruptions in vital influencers of their well-being, such as existing familial and social support and resources, psychological resilience, and heightened financial constraints, potentially leading to a decline in their environment-related QoL [21]. However, a previous comparative assessment of QoL in patients with different chronic diseases in Iran found that chronic respiratory diseases had an overall low QoL compared with other chronic diseases [22]. These differences could be explained by subject-specific and regional variations in the distribution of various determinants of QoL in patients with a chronic disease [8].

Our analysis revealed that patients who recovered from COVID-19 with one to two chronic diseases had statistically significantly lower scores in the physical health, psychological, and social relationship domains. The domain-specific rating was even lower in participants with three or more chronic diseases. Studies on patients with various chronic conditions have shown that coexisting chronic illnesses, risky health behaviors, depressive symptoms, sleeplessness, and cognitive impairment are all associated with decreased QoL [8]. On the other hand, chronic diseases can exacerbate disease severity, provide a dire prognosis, and increase fatality in patients with COVID-19 [23]. Therefore, the cumulative impact of COVID-19 plus chronic disease might have led to our subjects’ lower QoL even after recovery. To put more focus on this, the average QoL rating (q1) was significantly lower in participants with chronic diseases (*P* <0.001). A decreased health satisfaction score (q2) was noted in participants with chronic disease, indicating that they were unhappy with their current health condition days to months after recovery. However, people with chronic diseases reported having a low QoL during the COVID-19 outbreak in Saudi Arabia [24], which can be partially explained by the potential difficulty of finding medical care for chronic conditions during the COVID-19 pandemic [20]. However, another study conducted in Ethiopia showed that COVID-19 substantially impaired the HRQoL of hospitalized patients with comorbidities [21], implying a combined impact of multimorbidity on the QoL of those afflicted. On the multivariable analysis, we also found that chronic diseases were independently associated with a significantly lower QoL score in nearly all four domains of patients who recovered from COVID-19. In addition, having three or more chronic diseases was more detrimental for QoL than having one to two diseases. Notably, this was achieved after adjusting for socio-demographic variables, smoking habits, and history of hospital admission during COVID-19 illness. Nonetheless, only a few reports have considered the impact of comorbidity on the QoL of patients with COVID-19 after recovery. One such study by Chen *et al.* observed that the QoL score was significantly lower in patients 1 month after discharge from the hospital, and the presence of CKD was associated with the highest impact on physical health [25].

An overall lower score was observed in the psychological domain than three other domains, emphasizing the higher psychological burden of the participants in our study. In Ethiopia, a study showed that suspected COVID-19 cases with comorbidities had a seven-fold increased risk of having a common psychological defect [26]. Patients with chronic illnesses, heart disease, cancer, diabetes, high blood pressure, stroke, cognitive impairments, and psychotic disorders frequently experience emotional distress, nervousness, rage, and perplexity [27,28]. These factors increase the likelihood of recurrence or death, which might have increased the psychological burden in our study participants.

We conducted the study in 3244 patients who recovered from COVID-19. According to our research, almost half (46.85%) of the participants over 40 years of age had one to two chronic diseases and around 15% had three or more chronic diseases. These findings correspond with the distribution of chronic diseases among the adult population of Bangladesh [29,30]. We also found that married, divorced, or widowed participants were more likely to have chronic diseases than single participants. This difference could be explained by the fact that the former group was more likely to be older than the latter [17]. Smoking is a universal risk factor of many chronic diseases [17],and, quite expectedly, current smokers had a significantly higher proportion of chronic diseases than past and never smokers in our study.

### Limitations and recommendation

This investigation had several limitations. Because of its cross-sectional nature, this study could not make individual comparisons before and after COVID-19 infection among patients. Because the study was done during the pandemic, recruitment of participants without COVID-19 for comparison was not possible either. Moreover, only six self-reported NCDs were addressed. Nevertheless, this was one of the earlier attempts to assess the impact of comorbid non-communicable conditions on the QoL of people who recovered from COVID-19. Future endeavors in research should focus on comparing COVID-19 survivors and uninfected individuals to gain a deeper understanding of the challenges faced by COVID-19 survivors in managing chronic conditions and maintaining QoL. Longitudinal studies tracking disease progression over time would provide valuable insights into the long-term effects of COVID-19 on health outcomes. These comparative analyses would help identify areas where COVID-19 survivors may require additional support or interventions to improve their health and well-being. In addition, expanding the range of comorbidities studied would offer a more comprehensive understanding of their combined effects on the QoL. A consecutive comparison of individual morbidities could then reveal the differences in QoL outcomes in COVID-19 survivors with varying comorbidity profiles. Based on our findings, the current study provides several recommendations for clinicians and policymakers, namely:

• Improving the QoL of patients who recovered from COVID-19 should be prioritized.
• Health services for chronic NCDs should be reinstated and strengthened as early as possible.
• In the coming years, emphasis should be given to mental health services to attenuate the psychological burden and prevent the potential outbreak of a “mental health pandemic.”

## Conclusion

In conclusion, persons who recovered from COVID-19 with a comorbidity had a lower QoL in physical, psychological, social, and environmental domains. An inverse relationship between the number of chronic diseases and QoL was observed. The highest impact was observed in the psychological domain. The presence of cancer and CKD was associated with the most reduced QoL among the patients who recovered from COVID-19. As our global health response and mitigation measures for the COVID-19 pandemic grow, it is critical to understand and incorporate the impact on QoL. Our research emphasized the importance of continued care for chronic diseases to bring back people who were affected by COVID-19 to live a quality life. As the pandemic wanes and people around the world try to revert to normal, governments will develop plans and policies for an easy transition to a new start.

## Funding

Th is research did not receive any specific grant from funding agencies in the public, commercial, or not-for-profit sectors.

## Ethics approval and consent to participate

This project received ethical approval (No: 2020/OR-NSU/IRB-No.0801) from North South University's ethical review committee/institutional review board. The interviewer took verbal informed consent from the study participant during the phone interview. The interviewer recorded the permission on a form linked to the questionnaire. All procedures were carried out according to the 1964 Helsinki Declaration and its amendments and related ethical standards. The verbal informed consent from each study participant during the phone interview was recorded on a form linked to the questionnaire.

## CRediT authorship contribution statement

**Md. Abdullah Saeed Khan:** Conceptualization, Formal analysis, Resources, Software, Writing – review & editing. **Koustuv Dalal:** Conceptualization, Formal analysis, Writing – review & editing. **Mehedi Hasan:** Data curation, Visualization, Writing – original draft. **Miah Md. Akiful Haque:** Validation, Writing – original draft. **Nusrat-E-Mozid:** Investigation, Software, Writing – original draft. **Mosharop Hossian:** Data curation, Writing – review & editing. **Tajrin Rahman:** Methodology, Writing – original draft. **Ramisha Maliha:** Methodology, Writing – original draft. **Archi Mutsuddi:** Investigation, Writing – original draft. **Md. Utba Rashid:** Project administration, Visualization, Writing – review & editing. **Mohammad Ali Hossain:** Project administration, Resources, Writing – review & editing. **Mohammad Hayatun Nabi:** Conceptualization, Validation, Resources. **Mohammad Delwer Hossain Hawlader:** Conceptualization, Validation, Supervision, Writing – review & editing.

## Declarations of competing interest

The authors have no competing interests to declare.
